# The Efficacy and Safety of Rebamipide Ophthalmic Suspension (OPC-12759) in Patients with Dry Eye Disease: A Systematic Review of Randomized Controlled Trials

**DOI:** 10.3390/jcm12227155

**Published:** 2023-11-17

**Authors:** Antonio Ballesteros-Sánchez, María Carmen Sánchez-González, Concepción De-Hita-Cantalejo, Estanislao Gutiérrez-Sánchez, Carlos Rocha-de-Lossada, José-María Sánchez-González

**Affiliations:** 1 Department of Physics of Condensed Matter, Optics Area, University of Seville, 41004 Seville, Spain; msanchez77@us.es (M.C.S.-G.); mhita@us.es (C.D.-H.-C.); jsanchez80@us.es (J.-M.S.-G.); 2 Department of Ophthalmology, Ophthalmologic Novovision Clinic, 30008 Murcia, Spain; 3 Department of Surgery, Ophthalmology Area, University of Seville, 41009 Seville, Spain; egutierrez1@us.es (E.G.-S.); carlosrochadelossada5@gmail.com (C.R.-d.-L.); 4 Qvision, Ophthalmology Department, VITHAS Almeria Hospital, 04120 Almeria, Spain; 5 Ophthalmology Department, VITHAS Malaga, 29016 Malaga, Spain; 6 Regional University Hospital of Malaga, Hospital Civil Square, 29009 Malaga, Spain

**Keywords:** rebamipide ophthalmic suspension, secretagogues, goblet cells, dry eye disease

## Abstract

The aim of this paper is to evaluate the efficacy and safety of Rebamipide (REB) ophthalmic suspension in dry eye disease (DED). A systematic review that only included full-length randomized controlled studies (RCTs) reporting the effects of REB ophthalmic suspension in three databases, PubMed, Scopus and Web of Science, was performed according to the PRISMA statement. The Cochrane risk of bias tool was used to analyze the quality of the studies selected. A total of seven studies were included in this systematic review. Although the overall risk of bias was low, most studies were sponsored by the manufacturer. REB ophthalmic suspension treatment achieved higher improvement than the control group in all reported variables. The mean differences between both groups were in favor of the REB group and were as follows: dry eye-related quality of life score (DEQS) −3.5 ± 2.9 points, tear film break-up time (TBUT) of 0.7 ± 0.6 s, Schirmer test (ST) without anesthesia of 0.3 ± 0.6 mm and total corneal fluorescein staining (tCFS) of −1.2 ± 0.7 points. Adverse events (AEs) were 5.2 ± 7.6% superior in the REB group, with an overall compliance > 95%. Therefore, REB ophthalmic suspension is a safe and effective treatment that could be recommended in patients with DED.

## 1. Introduction

Dry eye disease (DED) is a multifactorial, complex and chronic disease of the ocular surface that causes discomfort, impaired vision and, in some cases, lesions in the cornea and conjunctiva [1]. DED affects up to 30% of adults over the age of 50, it is more frequent in women and its prevalence increases with age [2]. DED is characterized by an increase in tear film osmolarity due to a malfunction of the tear functional unit (LFU) [3,4], which comprises the lacrimal glands, ocular surface and eyelids, as well as the sensory and motor nerves that connect these components [4].

In recent years, DED treatments have been aimed at compensating for the deficiency in the affected tear layer, stimulating secretion and treating the underlying cause of DED [5]. Rebamipide (REB) ophthalmic suspension has recently been introduced into the market for the treatment of DED due to its mucin-secreting activity [6]. It is a quinolinone derivative and was originally developed for the treatment of gastritis or gastric ulcers [7]. It works by increasing the levels of prostaglandins E2 (PGE2) and prostacyclin (PGI2) in gastric tissues, favors the formation of gastric mucin and eliminates free radicals [8]. The ocular surface mucins provide defenses that protect it from the external environment and are also responsible for maintaining moisture [9]. Mucines are distinguished according to the subtype; secretory mucins (SM), such as MUC5AC, which are part of the mucosal layer of the tear film and are secreted by goblet cells [10]; and membrane-associated mucins (MAM), such as MUC1, MUC4 and MUC16 that are present in the tear film apical surface of the cornea and conjunctiva epithelium [11]. Both SM and MAM cover the ocular surface, providing a physical barrier against bacteria and viruses and possible trauma [11,12]. The lack of mucin, depending on the degree, can cause a series of pathologies from mild DED to more severe pathologies of the ocular surface [13].

REB ophthalmic suspension promotes the production of mucin-like glycoproteins in human corneal epithelial cells, increasing the expression levels of MUC1, MUC4, MUC-5AC and MUC16 via signals involved in epidermal growth factor receptor activation [14,15]. In addition, this substance also increases the number of goblet cells [16] and reduces corneal inflammation [17,18,19]. There are many studies that show its effectiveness in the treatment of DED. REB ophthalmic suspension (1% and 2%) has shown significant improvements in corneal and conjunctival staining, as well as tear film break-up time (TBUT) [20,21,22,23,24]. Igarashi et al. [25] reported an improvement in DED symptoms and signs among contact lens (CL) wearers. In addition, preoperative REB ophthalmic suspension treatment has been recommended in patients with DED to specify the calculation of the intraocular lens (IOL) [26], and it has been shown how the symptoms caused by tear instability and corneal irregularity also improve during the postoperative period after its administration [27].

The purpose of this systematic review is to assess the quality and methodology used in research, examining the efficacy and safety of REB ophthalmic suspension in patients with DED, as well as its potential influence on conjunctival goblet cells.

## 2. Materials and Methods

### 2.1. Data Sources and Search Strategy 

This systematic review was performed according to the Preferred Reporting Items for Systematic Reviews and Meta-Analyses (PRISMA) [28,29]. We identified 342 articles published before 30 July 2023 through the following databases: PubMed, Scopus and Web of Science. The data search strategy with Boolean operators was as follows: (rebamipide OR rebamipide ophthalmic suspension OR secretagogues) AND (dry eye disease OR DED OR evaporative dry eye OR EDE OR aqueous-deficient dry eye OR ADDE OR meibomian gland dysfunction OR MGD). The references of the retrieved articles were reviewed to identify other related studies if they met the inclusion criteria. This review was not registered in PROSPERO.

### 2.2. Study Selection 

The search strategy identified 342 articles, which were considered and analyzed. The DistillerSR software, version 9.0 (DistillerSR Inc., Ottawa, ON, Canada) was used to remove duplicate studies [30]. The remaining studies underwent additional screening stages. In the title and abstract screening, studies unrelated to the topic were excluded from the review. In addition, studies that did not include REB ophthalmic suspension treatment were also excluded from the review during the full-text screening. These studies were reviewed by two researchers who selected them according to the inclusion and exclusion criteria. The inclusion criteria included human studies, full-length original articles and prospective randomized controlled studies (RCTs). The exclusion criteria included unindexed journals and non-English publications. In addition, the country in which the study was performed, the follow-up period, the sample size or results of the studies were not considered for exclusion.

### 2.3. Quality Assessment and Data Extraction

The data from each study were collected and summarized independently in tables designed by two researchers. The following information was obtained from each article: (1) author and date of publication (year); (2) study design; (3) mean follow-up of all patients in the whole procedure (expressed in months); (4) number of patients; (5) mean age of the patients (expressed in years); (6) patient sex (male/female), (7) number of eyes involved; (8) inclusion criteria of the studies; (9) study group intervention; (10) control group intervention; (11) REB ophthalmic suspension posology; and (12) conflicts of interest. Regarding the results of the studies, the following data were collected: (13) dry eye-related quality of life score (DEQS, values from 0 to 100); (14) TBUT with fluorescein (expressed in seconds, s), which was evaluated by slit-lamp as the time from normal blinking to the first appearance of a dry spot in the tear film; (15) Schirmer test without anesthesia (ST, expressed in millimeters, mm) [31]; (16) total corneal fluorescein staining (tCFS), which was evaluated with the national eye institute grading scale (values from 0 to 15) [32] or the van Bijsterveld grading scale (values from 0 to 9) [33]; (17) ocular and non-ocular adverse events (AEs) (expressed as percentages); (18) compliance with REB ophthalmic suspension treatment; and finally, (19) authors judgment expressed by commenting in favor or against of REB ophthalmic suspension treatment. Baseline and last visit values for all these variables were collected in the REB and control groups.

Data synthesis was performed according to the Cochrane guideline for synthesis without meta-analysis (SWiM) [34]. Intra-group clinical outcomes were defined as “Last visit (LV)–Baseline (B) differences”. Inter-group clinical outcomes were defined as “REB group _(LV–B)_–control group _(LV–B)_ differences”. Mean ± standard deviation (SD) for each variable was calculated to report intra-group and inter-group clinical outcomes. The literature that remained after full-text screening was examined to assess the quality of the studies. To avoid the risk of bias, two dependable researchers created a synopsis based on the Cochrane risk of bias tool [35], which includes the following items: (1) random sequence generation, (2) allocation concealment, (3) blinding of participants and personnel, (4) blinding of outcome assessment, (5) incomplete outcome data, (6) selective reporting and (7) other sources of bias. A third nonblinded researcher decided the quality of the studies when disagreements occurred between the two researchers.

## 3. Results

### 3.1. Study Characterisitcs

**Figure 1:**
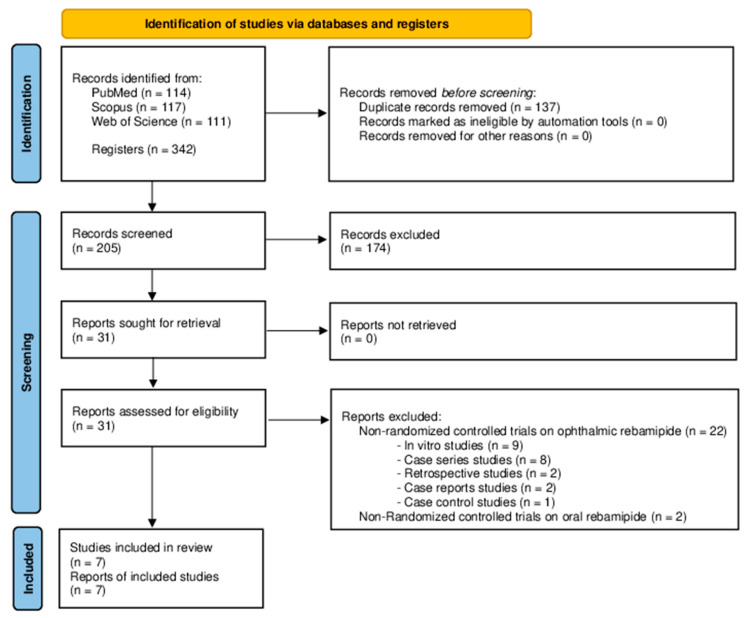
Flowchart study selection process according to the PRISMA statement.

The study selection process of this systematic review is presented with a flowchart diagram in Figure 1. The design of the included studies was prospective RCTs published between 2012 and 2023. This systematic review included 846 eyes from 741 patients with a mean age of 54.5 ± 12.1 years. The sex distribution was 151 males (20.4%) and 590 females (79.6%). Patient follow-up, expressed in months, ranged from 1 month to 3 months, with a mean follow-up of 1.3 ± 0.7 months. Regarding study group intervention, six studies used REB ophthalmic suspension alone (Otsuka Pharmaceutical Co., Tokyo, Japan) [20,21,22,33,36,37], while one study combined this treatment with diclofenac (Nitten Pharmaceutical Co., Nagoya, Japan) [38]. Control groups received different interventions, such as placebo [21], artificial tears [20,22,36], diclofenac [38] and diquafosol (DQS) ophthalmic solution (Santen Pharmaceutical Co., Osaka, Japan) [33,37]. Four studies had conflicts of interest by the authors (supported by Otsuka Pharmaceutical Co., Tokyo, Japan) [20,21,33,38]. More detailed characteristics of the studies are listed in Table 1.

### 3.2. Outcomes

Regarding efficacy outcomes, three studies reported dry eye symptom outcomes [20,21,22], of which two studies used the DEQS questionnaire [20,21] while one study used the OSDI questionnaire [22]. Six studies reported dry eye sign outcomes, of which all evaluated TBUT [20,21,33,36,37,38], three studies assessed ST [20,21,36] and five studies evaluated tCFS [20,21,33,36,37]. Regarding safety outcomes, three studies evaluated AEs [20,21,22], of which two studies reported ocular and non-ocular AEs [20,21], while one study only reported non-ocular AEs [22]. Intra- and inter-group clinical outcomes are presented in Table 2. Regarding the REB group, all results achieved an improvement, with a mean DEQS of −17.6 ± 6.2 points, mean TBUT of 0.8 ± 1.7 s, mean ST of 1.6 ± 1.3 mm and mean tCFS of −2.8 ± 1.1 points. Fewer improvements were achieved in the control group with a DEQS, ST and tCFS of −14.2 ± 9.1 points, 1.3 ± 0.8 mm and −1.6 ± 0.9 points, respectively. However, TBUT remained unchanged with a value of 0.02 ± 1.5 s. Regarding inter-group clinical outcomes, all the results were in favor of the REB group with a DEQS, TBUT, ST and tCFS of −3.5 ± 2.9 points, 0.7 ± 0.6 s, 0.3 ± 0.6 mm and −1.2 ± 0.7 points, respectively. In addition, the REB group also achieved higher improvements compared to patients who received DQS ophthalmic solution as control, with a mean DEQS of −3.5 ± 2.9 points, mean TBUT of 0.6 ± 0.1 s and mean tCFS of −0.6 ± 0.4 points.

Ocular and non-ocular AEs are presented in Table 3. The most common ocular AEs were visual impairment (6.5 ± 2.2%), conjunctival hemorrhage (5.8 ± 1.3%) and eye irritation (2.6 ± 1.7%), while non-ocular AEs were dysgeusia (23.6 ± 4.3%), nasopharyngitis (22.8 ± 2.6%) and white blood cell decrease (5.8 ± 3.3%). In addition, patients who received REB ophthalmic suspension reported that ocular AEs were 2.4 ± 0.5% lower than the control group. However, non-ocular AEs were 12.7 ± 1.4% superior in the REB group compared to the control group. Overall compliance with REB ophthalmic suspension treatment was 96.8 ± 3.4%.

**Table 1:** Summary of included RCTs.

Author (Date)	Design	F/U ^1^	Patients	Age ^2^	Sex (F/M)	Eyes	Inclusion Criteria	Intervention	Control	Posology ^3^ (TG/CG)	CoI
Kinoshita et al. [21] 2012	MT DM	1	308	55.2 [20 to 86]	269/39	308	DED	REB (1%) REB (2%)	Placebo	4/4	Yes
Kinoshita et al. [20] 2013	MT SM	1	188	56.6 ± 17.4	163/25	188	DED	REB (2%)	AT (SH 0.1%)	4/6	Yes
Igarashi et al. [36] 2015	MN	1	25	34.1 ± 10.8	22/3	50	DED refractive surgery	REB (2%)	AT (NR%)	4/4	No
Kato et al. [38] 2017	MT SM	1	33	71.1 ± 6.8	23/10	33	DED cataract surgery	REB (2%) Diclofenac (0.1%)	Diclofenac (0.1%)	4/3	Yes
Kobashi et al. [37] 2017	MN	1	40	70.4 ± 15.8	19/21	40	DED PK	REB (2%)	DQS (3%)	4/4	No
Shimazaki et al. [33] 2017	MT UM	1	67	39.9 ± 9.6	47/20	67	DED	REB (2%)	DQS (3%)	4/6	Yes
Jain et al. [22] 2023	MN	3	80	NR [18 to 75]	47/33	160	DED	REB (2%)	AT (CMC 0.5%)	4/4	No

AT, artificial tears; CMC, carboxymethylcellulose; CoI, conflict of interest; DQS, diquafosol ophthalmic solution; DM, double-masked; DED, dry eye disease; F, female; F/U, follow-up; M, male; MN, monocentric; MT, multicenter; NR, not reported; PK, penetrating keratoplasty; REB, rebamipide ophthalmic suspension; RCTs, randomized controlled trials; SH, sodium hyaluronate; SM, single-masked; TG, treatment group. ^1^ Expressed as months; ^2^ expressed as mean ± SD or range [IQR]; ^3^ number of drops administered per day in each eye.

**Table 2:** Intra-group and inter-group differences outcomes.

Author (Date)		Rebamipide Group				Control Group				Inter-Group Differences ^2^				F/A
		DEQS (0–100)	TBUT, s	ST, mm	tCFS	DEQS (0–100)	TBUT, s	ST, mm	tCFS	DEQS (0–100)	TBUT, s	ST, mm	tCFS	
Kinoshita et al. [21] 2012	Baseline	NR	2.8 ± 0.2	2.5 ± 0.3	7 ± 0.2	NR	2.9 ± 0.2	2.4 ± 0.2	6.9 ± 0.2	**-**	0.5 *	**0.5**	−1.8 *	F
	Last visit	NR	3.5 ± 0.3	3.5 ± 0.2	3.4 ± 0.1	NR	3.1 ± 0.3	2.9 ± 0.3	5.1 ± 0.2					
	**Difference** ^1^	**-**	0.7 *	**1**	−3.6 *	**-**	**0.2**	**0.5**	**−1.8**					
Kinoshita et al. [20] 2013	Baseline	NR	2.7 ± 0.1	2.5 ± 0.2	7 ± 0.2	NR	2.5 ± 0.1	2.3 ± 0.2	7 ± 0.2	**-**	**0.2**	**−0.5**	−0.8 *	F
	Last visit	NR	3.5 ± 0.1	3 ± 0.2	3.3 ± 0.3	NR	3.1 ± 0.1	3.3 ± 0.3	4.1 ± 0.8					
	**Difference** ^1^	**-**	**0.8**	**0.5**	−3.7 *	**-**	**0.6**	**1**	**−2.9**					
Igarashi et al. [36] 2015	Baseline	NR	2.2 ± 0.7	11.4 ± 9	4.3 ± 1	NR	2.7 ± 0.8	13.7 ± 11.9	4.1 ± 1.3	**-**	**2.2**	**1**	**−2.1**	F
	Last visit	NR	4.5 ± 1.7	14.9 ± 7.4	1.9 ± 1	NR	2.8 ± 0.8	16.2 ± 11.7	3.6 ± 1.2					
	**Difference** ^1^	**-**	2.3 *	3.5 *	−2.4 *	**-**	**0.1**	**2.5**	**−0.5**					
Kato et al. [38] 2017	Baseline	NR	6.9 ± 2.2	NR	NR	NR	7.4 ± 2.7	NR	NR	**-**	**0.5**	**-**	**-**	F
	Last visit	NR	4.2 ± 3.1	NR	NR	NR	4.2 ± 2.9	NR	NR					
	**Difference** ^1^	**-**	−2.7 *	**-**	**-**	**-**	−3.2 *	**-**	**-**					
Kobashi et al. [37] 2017	Baseline	28.8 ± 22	3.3 ± 1.4	NR	5.1 ± 3	23.3 ± 21	3.9 ± 1.5	NR	4.5 ± 3	**−6.4**	**0.6**	**-**	**−1**	F
	Last visit	17.3 ± 19	5.9 ± 1.5	NR	1.7 ± 1.6	18.2 ± 20	5.9 ± 1.6	NR	2.1 ± 2					
	**Difference** ^1^	**−11.5**	2.6 *	**-**	−3.4 *	**−5.1**	2 *	**-**	−2.4 *					
Shimazaki et al. [33] 2017	Baseline	48.6 ± 18	3.2 ± 0.8	15.2 ± 10.7	1.6 ± 1	44.7 ± 20	3.4 ± 1	13.7 ± 9.5	1.2 ± 0.7	**−0.6**	**0.5**	**-**	**−0.2**	F
	Last visit	24.8 ± 19	4.2 ± 0.8	NR	0.8 ± 0.4	21.5 ± 17	3.9 ± 0.9	NR	0.6 ± 0.6					
	**Difference** ^1^	−23.8 *	1 *	**-**	−0.8 *	−23.2 *	**0.5**	**-**	**−0.6**					
Jain et al. [22] 2023	Baseline	NR	NR	NR	NR	NR	NR	NR	NR	**-**	**-**	**-**	**-**	F
	Last visit	NR	NR	NR	NR	NR	NR	NR	NR					
	**Difference** ^1^	**-**	**-**	**-**	**-**	**-**	**-**	**-**	**-**					

DEQS, dry eye-related quality of life score; ST, Schirmer test with anesthetic; TBUT, tear film break-up time; tCFS, total corneal fluorescein staining. ^1^ Defined as Last visit–Baseline; ^2^ defined as (Rebamipide group _Last visit–Baseline_)–(control group _Last visit–Baseline_); * statistical significance level with *p* value < 0.05.

**Table 3:** AEs inter-group differences.

Author (Date)		Rebamipide Group	Control Group	Inter-Group Differences ^1^
Kinoshita et al. [21] 2012	Ocular AEs, %	5.9	8.7	**−2.8**
	Non-ocular AEs, %	20	8.7	**11.3**
Kinoshita et al. [20] 2013	Ocular Aes, %	7.5	9.4	**−1.9**
	Non-ocular AEs, %	16.1	2.1	**14**
Igarashi et al. [36] 2015	Ocular AEs, %	NR	NR	-
	Non-ocular AEs, %	NR	NR	-
Kato et al. [38] 2017	Ocular AEs, %	NR	NR	-
	Non-ocular AEs, %	NR	NR	-
Kobashi et al. [37] 2017	Ocular AEs, %	NR	NR	-
	Non-ocular AEs, %	NR	NR	-
Shimazaki et al. [33] 2017	Ocular AEs, %	NR	NR	-
	Non-ocular AEs, %	NR	NR	-
Jain et al. [22] 2023	Ocular AEs, %	NR	NR	-
	Non-ocular AEs, %	10	NR	-

AEs, adverse events. ^1^ Defined as (Rebamipide group)–(control group).

### 3.3. Risk of Bias

**Figure 2:**
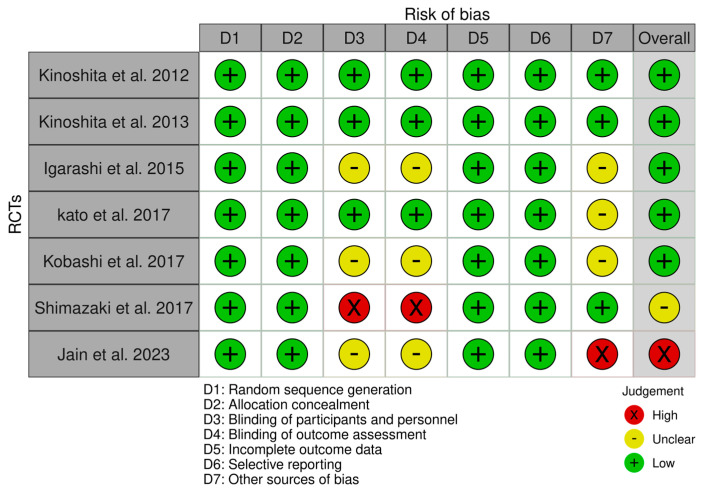
Risk of bias summary of the included studies with traffic light plot. The traffic lights represent the author’s risk of bias judgment in each domain (D) used to assess the quality of the studies.

**Figure 3:**
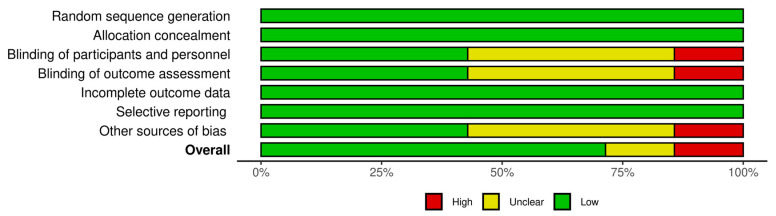
Overall risk of bias summary of the domains with bar plot. Bars represent the overall author’s risk of bias judgment in each domain presented as percentage.

The risk of bias summary is presented in Figure 2. Risk of bias assessment was classified into three evidence-level groups: (1) studies with a low risk of bias (Kinoshita et al. (2012) [21], Kinoshita et al. (2013) [20], Igarashi et al. [36], Kato et al. [38] and Kobashi et al. [37]), (2) studies with an unclear risk of bias (Shimazaki et al. [33]), and (3) studies with a high risk of bias (Jain et al. [22]). The overall risk of bias summary of the domains used in each study is shown in Figure 3. The items used to assess the risk of bias showed an overall low risk of bias, which was close to 75%. Therefore, no study was excluded due to the risk of bias. The Robvis tool (NIHR, Bristol, UK) was used to perform risk of bias assessment figures [39].

## 4. Discussion

Tear film hyperosmolarity is considered the trigger for the ocular surface inflammatory mechanisms, which lead to the DED symptoms and signs [4,40,41]. Different DED treatments based on eye drops have been proposed, such as tear substitutes [42,43], cyclosporine [44,45,46] and autologous serum [47,48]. However, compliance with these treatments has been shown to be suboptimal [49,50]. Therefore, there is an unmet need for new treatment options that improve tear film quality and restore ocular surface homeostasis while achieving satisfactory compliance [51]. This systematic review aimed to report the efficacy and safety of REB ophthalmic suspension treatment as a novel mucin secretagogue for patients with DED.

### 4.1. REB Ophthalmic Suspension Efficacy

In this systematic review, most studies reported DED symptoms with the DEQS questionnaire. This questionnaire has been validated for evaluating the multifaceted effect of DED on the patient’s daily life, and it is significantly correlated with the OSDI questionnaire [52,53], which is the most widely used for DED studies [31]. Kobashi et al. [37] and Shimazaki et al. [33] reported that patients who received REB ophthalmic suspension treatment achieved a DEQS score improvement of −3.5 ± 2.9 points compared to the control group. The main DED symptoms reported were foreign body sensation, blurred vision and photophobia, which were significantly reduced after REB ophthalmic suspension treatment compared to the control group [20,21,36]. The effects of REB ophthalmic suspension on tear film stability and volume, as well as on damage to the ocular surface, were evaluated by TBUT, ST and tCFS, respectively. Kinoshita et al. (2012) [21], Kinoshita et al. (2013) [20], Igarashi et al. [36], Kato et al. [38], Kobashi et al. [37] and Shimazaki et al. [33] reported that REB ophthalmic suspension treatment achieved a TBUT improvement of 0.7 ± 0.6 s compared to the control group. In addition, Kinoshita et al. (2012) [21], Kinoshita et al. (2013) [20] and Igarashi et al. [36] also reported an ST improvement of 0.3 ± 0.6 mm after REB ophthalmic suspension treatment compared to the control group. It is important to mention that TBUT and ST were evaluated with fluorescein and without anesthesia, respectively. Therefore, the invasiveness of these tests may influence the results by masking the effects of REB ophthalmic suspension on DED [31]. Kinoshita et al. (2012) [21], Kinoshita et al. (2013) [20], Igarashi et al. [36], Kobashi et al. [37] and Shimazaki et al. [33] reported that REB ophthalmic suspension treatment achieved a tCFS improvement of −1.2 ± 0.7 points compared to the control group, which was significant after 2 weeks of treatments. This result may be compared with other drugs that are used in current DED therapies, such as sodium hyaluronate and cyclosporine, which have been demonstrated to achieve a significant improvement in tCFS after 4 weeks [54] and 4 months [55], respectively. It is also important to mention that the results obtained by Kato et al. [38] and Kobashi et al. [37] seem to be slightly lower compared to the remaining studies. This may be explained by both studies, including patients > 70 years, since it has been shown that the severity of DED increases with age [2].

The effects of REB ophthalmic suspension have also been studied in patients with DED undergoing ocular surgery. Teshigawara et al. [27] reported that preoperative administration of REB ophthalmic suspension achieved higher DED symptoms and signs of relief compared to sodium chloride solution, which may help to increase patient satisfaction after ocular surgery [26]. Similar results were also reported by Kobashi et al. [37] after penetrating keratoplasty, with significant improvements in TBUT, ST and tCFS. In addition, REB ophthalmic suspension treatment also seems to significantly reduce intraocular light scattering [36] and improve goblet cell density in the postoperative period [37]. All these findings may be explained by its mechanism of action. REB ophthalmic suspension treatment promotes the production of mucin-like glycoproteins in human corneal epithelial cells, increasing the number of goblet cells [16], as well as the expression levels of MUC1, MUC4, MUC-5AC and MUC16 [14,15]. It is thought that disturbance of ocular surface mucin is one of the main causes of tear film instability [4]. Therefore, it is hypothesized that increased mucin levels after REB ophthalmic suspension administration improve ocular surface wettability [14,15,16], which leads to a more uniform and stable tear film, thus alleviating DED symptoms and signs [20,21,22,33,37].

### 4.2. REB Ophthalmic Suspension Safety

Kinoshita et al. (2012) [21], Kinoshita et al. (2013) [20] and Jain et al. [22] reported AEs after REB ophthalmic suspension treatment. Non-ocular AEs were more common than ocular AEs, which may be expected due to the bitter taste associated with REB [20]. Dysgeusia, nasopharyngitis and white blood cell decrease were the most reported non-ocular AEs, while ocular AEs included visual impairment, conjunctival hemorrhage and eye irritation. However, they were mild in severity and recovered without any treatment, suggesting the satisfactory tolerability of REB ophthalmic suspension. This tolerability may be due to REB ophthalmic suspension does not contain preservatives [20,22], such as benzalkonium chloride (BAK), which elevates the concentrations of inflammatory markers in ocular tissues, leading to corneal epithelium and conjunctival goblet cells apoptosis [56,57,58]. Compliance with REB ophthalmic suspension treatment may be put in context with other DED therapies. Topical cyclosporine and lifitegrast studies have reported that burning is the main ocular AEs with an overall compliance of 80.2% [45,55,59] and 91.1 [60,61,62], respectively. Varenicline nasal spray, which has recently been approved for DED treatment [63], seems to reduce the common AEs of topical ocular application modalities by achieving an overall compliance of 95.4% [64,65,66]. However, this systematic review has reported an overall compliance with REB ophthalmic suspension treatment of 96.8 ± 3.4%. This result suggests REB as a potentially safe option for patients with DED. In addition, new REB formulations are under research to further enhance pent compliance [67].

### 4.3. REB Ophthalmic Suspension vs. DQS Ophthalmic Solution

DQS is a dinucleotide P2Y2 receptor agonist that has also recently been approved for the treatment of DED [68]. This dinucleotide has been demonstrated to upregulate the expression levels of MUC1, MUC4, MUC-5AC and MUC16 [69], which are responsible for the ocular surface epithelium wettability [9]. There are many studies that show the effectiveness of DQS ophthalmic solution in the treatment of DED. DQS ophthalmic solution has shown significant improvements in DED symptoms and signs, such as TBUT, ST and ocular surface staining [70,71,72]. Ogami et al. [73] also reported improvements in DED symptoms and signs among CL wearers after DQS ophthalmic solution administration. In addition, recent studies have reported the influence of DQS ophthalmic solution on conjunctival goblet cell density [74], as well as its potential usefulness for the treatment of obstructive meibomian gland dysfunction [75,76]. Although these findings suggest DQS as a novel treatment for DED with promising results, this systematic review has reported that REB ophthalmic suspension treatment achieves slightly better results than DQS ophthalmic solution. This may be explained by the anti-inflammatory and antioxidant unique properties of REB [77], but further research is needed.

In addition, REB ophthalmic suspension and DQS ophthalmic solution have also been demonstrated to improve the tear film parameters and clinically reduce DED symptoms and signs in patients with diabetes [78,79]. This highlights the need for further research into the off-label use of these treatments, especially in systemic and ocular diseases that are associated with DED.

### 4.4. Strengths and Limitations

The main strength of this systematic review is the results obtained because all studies included were RCTs with an overall low risk of bias. Regarding studies’ methodologies, although the posology was practically the same, the inclusion criteria and interventions in both groups were different between studies. Therefore, this heterogeneity in the methodologies makes it difficult to perform a meta-analysis, which shows that new studies with remarkably similar methodologies are needed to perform a meta-analysis to confirm the results obtained in this study. In addition, it would also be interesting to perform a meta-analysis that analyzes different DED treatments in parallel in order to better understand the effectiveness and safety of different available treatment options.

This study has several limitations that may have influenced the results. First, the relatively short follow-up period of the studies included. Therefore, there is a need for larger, well-designed, strictly blind, multicenter RCTs evaluating the long-term efficacy and safety of REB ophthalmic suspension treatment in DED at different concentrations, particularly in patients with Sjogren’s syndrome, which is the main cause of aqueous-deficient dry eye [80]. In addition, it would be interesting to compare these results with other secretagogues, such as DQS ophthalmic solution. All this information could be helpful in establishing the most effective and safe secretagogue in DED, as well as its effective daily dose and concentration. Second, the influence of TBUT with fluorescein and ST without anesthesia on the interpretation of the results. Thus, further studies analyzing tear film by objective and non-invasive tests, such as non-invasive tear film break-up time (NIBUT), tear meniscus height (TMH) and tear meniscus area (TMA), are needed to avoid the influence of traditional tests on tear film and provide more accurate results [81]. Finally, it is important to mention that most of the studies included in this systematic review were supported by Otsuka Pharmaceutical Co., Tokyo, Japan. Hence, there is an unmet need for further non-industry-funded studies.

## 5. Conclusions

In conclusion, this systematic review has demonstrated that REB ophthalmic suspension treatment achieves better results than the control groups. REB ophthalmic suspension treatment improves DED symptoms and signs, reporting high compliance due to its tolerability and minimal AEs. In addition, this treatment also seems to improve the intraocular light scattering and conjunctival goblet cell density in the postoperative period. Therefore, REB ophthalmic suspension is an effective and safe treatment that may be recommended for patients with DED. This treatment may be represented as a novel candidate for DED treatment due to its mucin secretagogue activity, but further RCTs are still needed.

## Data Availability

The data presented in this study are available on request from the corresponding author. The data are not publicly available due to information that could compromise the privacy of research participants.
